# A new species and a revised key of the genus *Thoradonta* (Orthoptera, Tetrigidae)

**DOI:** 10.3897/zookeys.607.9056

**Published:** 2016-07-27

**Authors:** Ling-Sheng Zha, Maoyin Sheng, Ting-Chi Wen, Kevin D. Hyde

**Affiliations:** 1 School of Karst Science, Guizhou Normal University, Guiyang, 550001, China; 2 School of Life Sciences, Huaibei Normal University, Huaibei, 235000, China; 3 Institute of Excellence in Fungal Research, and School of Science, Mae Fah Luang University, Chiang Rai, 57100, Thailand; 4 National Engineering Research Center for Karst Rocky Desertification Rehabilitation, Guiyang, 550001, China

**Keywords:** Ecology, habits, morphological variation, Scelimeninae, taxonomy

## Abstract

A new species of the genus *Thoradonta* (Orthoptera, Tetrigidae), *Thoradonta varispina* Zha & Sheng, **sp. n.**, was found in Lengshuihe Nature Preserve, Jinsha, Guizhou, China. It is introduced with a description and photographs and compared with similar taxa. Ecology, habits, and morphological variation of the new species are discussed and illustrated. Generic characteristics of *Thoradonta* are updated and an updated key to all known species of *Thoradonta* is given.

## Introduction

The genus *Thoradonta* Hancock belongs to Scelimeninae, Tetrigidae, type species *Thoradonta dentata* Hancock. To date it includes 21 known species worldwide, distributed in subtropical and tropical Asia (China, Bengal, Hong Kong, India, Indonesia, Malaysia, Myanmar, Nepal, Singapore, Sri Lanka, the Philippines, and Vietnam) and equinoctial Africa (Zha et al. 2016b).

During investigation of species diversity in Lengshuihe Nature Preserve, Jinsha County, Guizhou Province, China during 3–10 Aug 2015, a new species of the genus *Thoradonta* was found, *Thoradonta varispina* Zha & Sheng, sp. n. A description and illustrations introduce the species, and it is compared with similar taxa. Ecology, habits, and morphological variation of body structure of the genus *Thoradonta* are discussed. Generic characteristics of *Thoradonta* are updated and an updated key to all known species of the genus is given.

## Material and methods

Specimens were photographed using a stereomicroscope (Keyence VHX-1000). Morphological terminology and measurement landmarks follow Shishodia (1991) and Zheng (2005). Measurements are given in millimeters (mm). Type specimens are deposited in the Specimen Room of the School of Life Sciences, Huaibei Normal University, Huaibei, Anhui, China.

## Taxonomy

### Thoradonta varispina

Taxon classificationAnimaliaOrthopteraTetrigidae

Zha & Sheng sp. n.

http://zoobank.org/57E266B7-4DF3-4656-AC37-7EAD7FDC0E38

Figs 1 , 2 , 3

#### Diagnosis.

*Thoradonta varispina* sp. n. is distinguished from *Thoradonta obtusilobata* Zheng, 1996 by the following characters: 1) vertex 1.8–2.0 times as wide as one eye (width of the widest part of an eye in dorsal view); 2) midkeel of pronotum not reaching anterior margin of pronotum; 3) upper margin of hind femur before antegenicular denticle with a small protrusion only, but not forms into 2–3 lamellae; 4) lower margin of hind femur entire, without protrusion; 5) third pulvillus of first segment of hind tarsus distinctly longer than first and second.

#### Description.

Female. Body size small, covered with numerous small granules and many nodules.

*Head.* Head not protruding over level of pronotal surface; vertex 1.8-2.0 times as wide as one eye, anterior margin straight, protruding but not surpassing anterior margins of eyes, lateral margins folded upwards but not surpassing top of eyes; median carina conspicuous and protruding in anterior half which is visible before eyes in profile, while obscure or absent in posterior half; vertex together with frontal ridge arcuate and protruding, distinctly concave between lateral ocelli, then strongly arched and protruding between antennae, longitudinal furrow between antennae slightly wider than diameter of first segment of antenna, margins of longitudinal furrow finely serrate. Antenna filiform, 17-segmented, inserted slightly below lower margins of eyes, length of longest segment (segment IV, male in VIII) 4.5 times its width. Eyes globose, protruding but not above level of pronotum; lateral ocellus situated slightly below middle of anterior margin of eye.

*Thorax.* Pronotum very coarse, covered with numerous small granules and many nodules; middle of anterior margin little concave; lateral keels of prozona erected, distal part higher than basal part, slightly contracted backward, sometimes distal part excessively contracted inward. Midkeel not reaching anterior margin of pronotum, otherwise nearly entire, in profile upper margin of pronotum distinctly undulate with wave peaks becoming lower backward. These sinusoidal waves lamellate and erected, first highest, with intumesced base, both sides of intumescence with a pair of big nodules at margins of pronotum; second lamella longest and undulate, on both sides pronotal disc distinctly concave followed by a pair of long oblique nodules, and lateral margins of pronotum distinctly folded upwards; the latter midkeel with 4–5 lamellae of intumesced base. Humeral angle obtusely angled; a pair of abbreviated carinae present between shoulders, slightly contracted forwards; pronotum slightly uplifted between shoulders; hind process of pronotum long cone-shaped, reaching (few specimens) or slightly surpassing (most specimens) apex hind femora, distal part slightly down-curved and apex sharp. Posterior angles of lateral lobes of pronotum laminate and expanded and extending outwards, apex varies distinctly in different individuals: upper lobe not produced, slightly produced or triangularly laterally produced; subtruncate behind which is margined with fine teeth (Fig. 3). Posterior margin of each lateral lobe has two concavities. Visible part of tegmina ovate, 2.7 times as long as wide, apex rounded. Hind wing not reaching top of hind process of pronotum, not reaching or reaching apex of hind femur. Margins of all femora with fine teeth; upper margin of fore femur slightly undulate, lower margin distinctly undulate; upper and lower margins of mid femur distinctly undulate; mid femur slightly wider than fore femur and visible part of tegmen, not narrowed or thicker from basal to distal area. Hind femur about 2.5 times as long as wide, rear of upper margin before antegenicular denticle slightly protruding; antegenicular denticle isolated and long triangular, its apex relatively sharp, genicular denticle fingered extending backward and apex obtuse. Hind tibia distally slightly wider than basally, outer side with 5–7 spines, inner side with 4–6 spines; first segment of hind tarsus 1.3–1.4 times longer than third, third pulvillus longer than first and second, apex of third pulvillus relatively obtuse, apices of first and second sharp.

*Abdomen.* Ovipositor: upper valvulae 3.3 times as long as wide, outer margins of upper and lower valvulae with saw-like teeth. Posterior margin of subgenital plate: narrowing backward; middle triangularly protruding, sometimes this protrusion folded inward, forming a basal concavity and a protrusion on both sides (Fig. 2d, e).

*Coloration.* Body dark brown. Antenna brown, distal segments darker than basal segments; hind wings black; for and mid femora and tibiae with 3 yellowish brown bands each, bands of all femora obscure; lower outside of hind femur black, center of inner side of hind femur dark brown; hind tibia with 2 long yellowish brown bands.

**Male.** Body size slightly smaller than female. Antenna 16-segmented. Fore femur nearly the same as that of female; mid femur distinctly wider than fore femur and visible part of tegmen, narrowing from basal to distal side, and basal part thicker than distal part. Subgenital plate short cone-shaped, apex bifurcate but not bidentate.

#### Measurements.

Length of body ♂6.2–7.8 mm, ♀7.5–9.0 mm; length of pronotum ♂6.1–7.0 mm, ♀7.5–8.7 mm; length of hind femur ♂3.5–4.2 mm, ♀4.7–5.4 mm.

#### Type material.

Holotype female, China, Guizhou, Jinsha County, Lengshuihe Natural Reserve, N27°54', E106°00', 650 m alt, 7 Aug. 2015, collected by Ling-Sheng ZHA. Paratypes: 27 males and 19 females, 500–800 m alt, 5–9 Aug. 2015, other data same as holotype.

**Figure 1.**
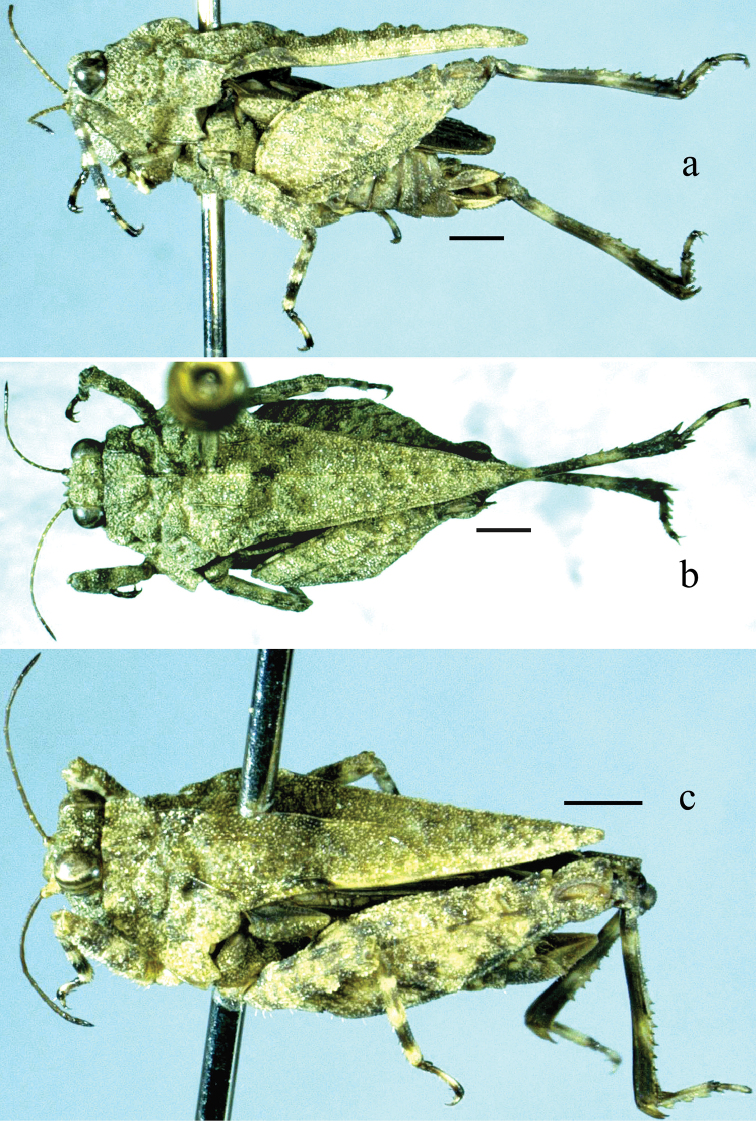
*Thoradonta varispina* sp. n.: **a–b** lateral and dorsal views of female **c** oblique view of male. Scale bars: 1.0 mm.

**Figure 2.**
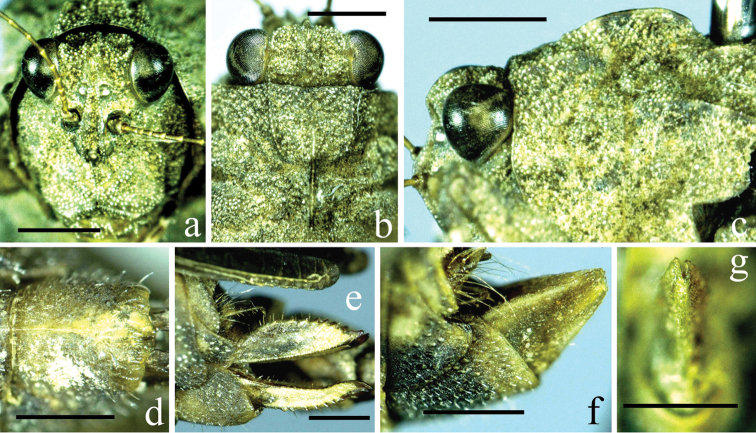
*Thoradonta varispina* sp. n.: **a** frontal view of female head **b** dorsal view of female head and anterior part of pronotum **c** lateral view of female head and anterior area of pronotum **d** ventral view of female subgenital plate **e** lateral view of female ovipositor **f** laterial view of male subgenital plate **g** posterior view of male subgenital plate. Scale bars **a–c**: 1.0 mm, **d–g**: 0.5 mm.

**Figure 3.**
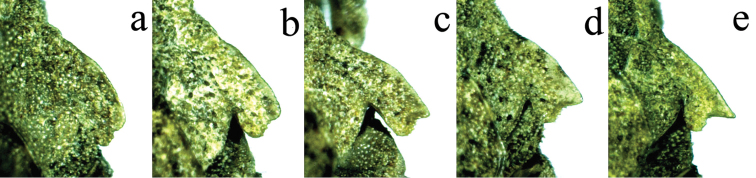
Outline of lateral lobes of pronotum with apex of posterior angle of different individuals of *Thoradonta varispina* sp. n..

#### Ecology and habits.

**Figure 4.**
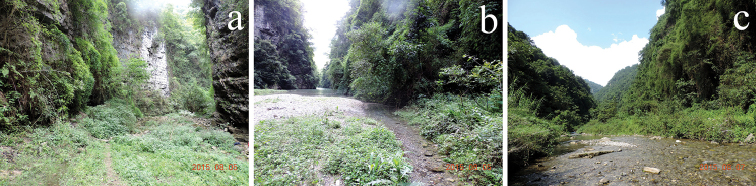
Habitats of *Thoradonta varispina* sp. n. (photographed in Lengshuihe Natural Reserve, Jinsha County, Guizhou, China).

All specimens of the new species were collected in humid, sandy, and stony environments alongside streams (Fig. 4). Body surfaces of most individuals are covered tightly by numerous sand grains. They move frequently in sunshine, and they feed on mosses, algae, lichens and all sorts of humus.

#### Etymology.

This new species epithet means spine of upper lobe of posterior angle of lateral lobe of pronotum varies in different individuals.

#### Distribution.

China (Guizhou).

### Key to species of the genus *Thoradonta* Hancock, with distributions

**Table:** 

1	Tegmen and wing invisible. Indonesia	***Thoradonta butlini* Blackith & Blackith**
–	Tegmina and wings visible	**2**
2	Upper lobe of posterior angle of lateral lobe of pronotum not produced or slightly produced, not spinose (Fig. 3)	**3**
–	Upper lobe of posterior angle of lateral lobe of pronotum produced conspicuously and spinose (Fig. 5)	**5**
3	Body length 10.79–12.81 mm; hind process of pronotum distinctly surpassing apex of hind femur (Fig. 6e). Nepal	***Thoradonta aspinosa* Ingrisch**
–	Body length 6.0–9.0 mm; hind process of pronotum shorter, only reaching or slightly surpassing apex of hind femur (Fig. 6b, c)	**4**
4	Vertex 1.5 times as wide as one eye; midkeel of pronotum reaching anterior margin of pronotum; upper margin of hind femur before antegenicular denticle with 2–3 lamellate protrusions, lower margin with a distinct protrusion. China	***Thoradonta obtusilobata* Zheng**
–	Vertex 1.8–2.0 times as wide as one eye; midkeel of pronotum not reaching anterior margin of pronotum; upper margin of hind femur before antegenicular denticle with a small protrusion only, lower margin without protrusion. China	***Thoradonta varispina* sp. n.**
5	Hind process of pronotum shorter, not reaching or reaching apex of hind femur (Fig. 6a, b)	**6**
–	Hind process of pronotum longer, surpassing apex of hind femur (Fig. 6c–e)	**9**
6	Hind process reaching apex of hind femur (Fig. 6b); lower lobe of posterior angle of lateral lobe of pronotum acutely produced (Fig. 5c). India, Bengal	***Thoradonta bengalensis* Shishodia**
–	Hind process not reaching apex of hind femur (Fig. 6a); lower lobe of posterior angle of lateral lobe of pronotum truncate or subtruncate	**7**
7	Wings shorter, not reaching apex of hind process; third pulvillus of first segment of hind tarsus longer than second (Fig. 5b). China, Hong Kong, India, Indonesia, Malaysia, Singapore, Sri Lanka	***Thoradonta nodulosa* (Stål)**
–	Wings longer, reaching apex of hind process; third pulvillus of first segment of hind tarsus equal to second in length	**8**
8	Upper lobe of posterior angle of lateral lobe of pronotum obliquely dentate (Fig. 5b). Malaysia	***Thoradonta dentata* Hancock**
–	Upper lobe of posterior angle of lateral lobe of pronotum acutely spinose, pointing laterally (Fig. 5a). Equinoctial Africa	***Thoradonta spinata* Hancock**
9	Body size stout; hind process of pronotum surpassing slightly beyond apex of hind femur (Fig. 6c)	**10**
–	Body size slender; hind process of pronotum surpassing far beyond apex of hind femur (Fig. 6d, e)	**13**
10	Anterior margin of vertex nearly as wide as posterior margin; spine of upper lobe of posterior angle of lateral lobe of pronotum pointing distinctly obliquely backward (Fig. 5b). China	***Thoradonta yunnana* Zheng**
–	Anterior margin of vertex distinctly narrower than posterior margin; spine of upper lobe of posterior angle of lateral lobe of pronotum pointing laterally or slightly obliquely backward	**11**
11	Spine very long (Fig. 5c); wings reaching apex of hind process of pronotum. China	***Thoradonta longispina* Zheng & Xie**
–	Spine shorter (Fig. 5a); wings not reaching apex of hind process of pronotum	**12**
12	Antenna inserted at the level of lower margins of eyes, the longest segment 4.0 times as long as wide; first segment of hind tarsus 1.75 times as long as third. China, India	***Thoradonta spiculoba* Hancock**
–	Antenna inserted decidedly below lower margins of eyes, the longest segment 6 times as long as wide; first segment of hind tarsus 1.3–1.4 times as long as third. Thailand	***Thoradonta spiculobaoides* Zha & Kang**
13	Spine of upper lobe of posterior angle of lateral lobe of pronotum pointing laterally (Fig. 5a, c)	**14**
–	Spine of upper lobe of posterior angle of lateral lobe of pronotum pointing obliquely backward (Fig. 5b)	**16**
14	Vertex 2.0 times as wide as one eye; wings not reaching apex of hind process (Figs 5c, 6c, d). India, Vietnam	***Thoradonta centropleura* Podgornaya**
–	Vertex not more than 1.5 times as wide as one eye; wings reaching or surpassing apex of hind process	**15**
15	Spine slender and longer (Fig. 5c); pronotum 2.4–3.0 times as long as posterior part of hind process which is beyond hind femur (Fig. 6e); wings surpassing apex of hind process. China	***Thoradonta longipenna* Zheng & Liang**
–	Spine shorter (Fig. 5a); pronotum 3.3–4.0 times as long as posterior part of hind process which is beyond hind femur (Fig. 6d); wings reaching apex of hind process. China	***Thoradonta transspicula* Zheng**
16	Vertex wider, 1.5–2.0 times as wide as one eye (Fig. 6d)	**17**
–	Vertex narrower, 1.18–1.35 times as wide as one eye	**21**
17	Wings longer, surpassing far beyond apex of hind process; disc of pronotum black. China	***Thoradonta nigrodorsalis* Zheng & Liang**
–	Wings shorter, not reaching or surpassing slightly beyond apex of hind process; pronotum and body in the same color	**18**
18	Pronotum not less than 5.0 times as long as posterior part of hind process which is beyond apex of hind femur	**19**
–	Pronotum not more than 4.0 times as long as posterior part of hind process which is beyond apex of hind femur	**20**
19	Lateral keels of prozona parallel; wings not reaching apex of hind process. China	***Thoradonta lancangensis* Zheng**
–	Lateral keels of pronoza distinctly contracted backward; wings surpassing apex of hind process. China	***Thoradonta dianguiensis* Deng, Zheng & Wei**
20	Antenna inserted between lower margins of eyes; lateral keels of pronoza parallel; humeral angle widely rounded. China, India	***Thoradonta lativertex* Günther**
–	Antenna inserted decidedly below lower margins of eyes; lateral keels of pronoza distinctly contracted backward; humeral angle obtusely angled. Thailand	***Thoradonta lativertexoides* Zha & Kang**
21	Vertex 1.25–1.35 times as wide as one eye; body surface smooth; length of posterior part of hind process which is beyond apex of hind femur 3.0–3.4 mm (Fig. 6e). The Philippines	***Thoradonta palawanica* Günther**
–	Vertex nearly as wide as one eye; body surface coarse; length of posterior part of hind process which is beyond apex of hind femur 1.5-2.0 mm (Fig. 6d). China, India, Myanmar, Thailand	***Thoradonta apiculata* Hancock**

**Figure 5.**
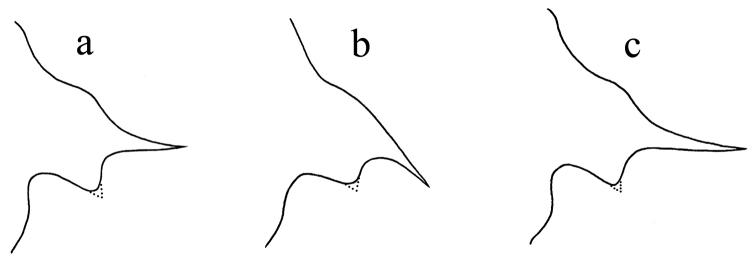
Variations of spine of posterior angle of lateral lobe of pronotum in the genus *Thoradonta*: **a** normal and pointing laterally **b** normal and oblique backward **c** long and pointing laterally.

**Figure 6.**
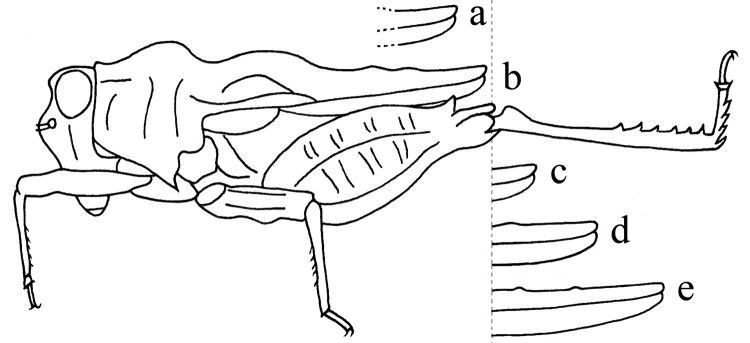
Variations of the length of pronotum in the genus *Thoradonta*: **a** hind process of pronotum doesn’t reach apex of hind femur **b** reaches apex of hind femur **c** slightly surpasses apex of hind femur **d** nearly reaches middle of hind tibia **e** nearly reaches apex of hind tibia.

## Discussion

Species of the genus *Thoradonta* generally live in humid and sandy places near streams, rivers, or ponds. They move frequently in sunshine, and they generally feed on mosses, algae, lichens and all sorts of humus. Though provided with developed hind wings they seldom really fly, instead their hind femora are well-developed, suitable for jumping when disturbed. Colors of their bodies are generally adapted to the soil of their habitats. Apart from generally coarse an uneven, body surfaces of most individuals were often tightly covered by numerous sand grains. We infer that they lay eggs in sandy soil, and most of their life time they may conceal their body in sandy soil (Zha et al. 2016a) to avoid bad environments such as low temperature, being preyed, rain, drought etc.; when temperature is high and light is good, they may crawl out from sandy soil for feeding and mating. Their small size and long-term living in sandy soil made them easily be preserved during evolution. Additionally, based on collecting times of all known adults (from beginning of April to end of November), we infer part or all species of the genus living outside the tropics may overwinter as adults (the genus from tropical countries do not hibernate at all).

According to *Thoradonta varispina* sp. n., and comparing with descriptions of 21 known species of the genus *Thoradonta* (Hancock 1909, 1915, Günther 1938, Zheng 1983, 1996, 2005, Blackith and Blackith 1987, Shishodia 1991, Zheng and Liang 1991, Podgornaya 1994, Ingrisch 2001, Deng et al. 2006, Zha et al. 2016b), generic characteristics of *Thoradonta* should be updated as follows.

Body size small. Vertex equal to or wider than one eye, frontal ridge distinctly protruding forward between antennae. Antenna filiform, inserted between or below lower anterior margins of eyes. Eyes globular and prominent, lateral ocellus situated in or slightly below middle of anterior margin of eye. Distal segments of maxillary palpus slightly compressed. Pronotal disc generally covered with many nodules; midkeel undulate, partially lamellate and erected before shoulders; pronotum slightly uplifted between shoulders; a pair of abbreviated carinae present between shoulders; lateral margins of pronotum behind humeral angles folded upwards; hind process of pronotum wedge-shaped, not reaching, reaching or surpassing apex of hind femur; posterior angle of lateral lobe of pronotum laminate and expanded and extending outwards, apex varies conspicuously: 1) upper lobe generally produced, spinose or acutely angled, extending laterally or obliquely backward; 2) lower lobe generally truncate, sometimes also produced and obtusely angled or acutely angled; 3) both upper and lower lobes not produced or produced inconspicuously, and apex truncate or subtruncate. Visible part of tegmina ovate, hind wing normal but invisible in *Thoradonta butlini*. First segment of hind tarsus generally longer than third.

Podgornaya (1994) indicated two forms of wings and pronotum (brachypterous and macropterous) as occurring in *Thoradonta spiculoba* specimens collected from Vietnam, as well as *Thoradonta apiculata* from Thailand reported by Storozhenko and Dawwrueng (2015) recently, while in *Thoradonta varispina* sp. n., though varying more or less it is indistinct. So we think the view that similar specimens with different lengths of both wings and pronotum in Tetrigidae are two different species is debatable, at least not so in *Thoradonta varispina* sp. n.. Notably for *Thoradonta varispina* sp. n., the apex of the posterior angle of the lateral lobe of pronotum varies conspicuously between individuals, but never forms into a spine, which is easily distinguished from other spinose species of the genus. The morphological variation of apex of lateral lobe of pronotum from spinose to truncate indicates that Scelimeninae is very close to Metrodorinae in phylogeny, and this finding would help studying taxonomy and evolution of these Tetrigidae insects.

## Supplementary Material

XML Treatment for Thoradonta varispina

## References

[B1] Blackith RE Blackith RM (1987) Tridactylids and Tetrigids (Orthoptera) from Sulawesi, Indonesia. Tijdschrift voor Entomologie 130: 1–10.

[B2] Deng WA Zheng ZM Wei SZ (2006) Two new species of Scelimenidae from Yunnan and Guangxi, China (Orthoptera: Tetrigoidea). Acta Zootaxonomica Sinica 31(2): 369–372. [In Chinese with English summary]

[B3] Günther K (1938) Revision der Acrydiinae (Orthoptera) II, Scelimenae spuriae. Stettiner Entomologische Zeitung 99: 117–148.

[B4] Hancock JL (1909) Further studies of the Tetriginae (Orthoptera) in the Oxford University Museum. Transactions of the Entomological Society of London 56(3/4): 387–426. doi: 10.1111/j.1365-2311.1909.tb02160.x

[B5] Hancock JL (1915) Indian Tetriginae (Acrydiinae). Records of the Indian Museum Calcutta 11: 80–82.

[B6] Ingrisch S (2001) Orthoptera of the Nepal expeditions of Prof. J. Martens (Mainz). Senckenbergiana biologica 81: 147–186.

[B7] Podgornaya LI (1994) Notes on the genus *Thoradonta* Hancock (Orthoptera: Tetrigidae). Proceedings of the Zoological Institute of the Russian Academy of Sciences, St. Petersburg 257: 51–54. [In Russian]

[B8] Shishodia MS (1991) Taxonomy and zoogeography of the Tetrigidae (Orthoptera: Tetrigoidea) of North Eastern India. Records of the Zoological Survey of India, Occasional Papers 140: 1–204.

[B9] Storozhenko SYu Dawwrueng P (2015) New and little-known pygmy grasshoppers (Orthoptera: Tetrigidae) from Thailand. Zootaxa 4052(5): 527–554. doi: 10.11646/zootaxa.4052.5.2 26701451 10.11646/zootaxa.4052.5.2

[B10] Zha LS Wen TC Kang JC Hyde KD (2016a) Records of *Hedotettix* and *Teredorus* in Thailand with the description of three new species (Orthoptera, Tetrigidae). ZooKeys 556: 83–95. doi: 10.3897/zookeys.556.6002 26877695 10.3897/zookeys.556.6002 PMC4740873

[B11] Zha LS Wen TC Kang JC Hyde KD (2016b) The genus *Thoradonta* in Thailand (Orthoptera: Tetrigidae: Scelimeninae) with description of two new species. Journal of Natural History 50(13/14): 833–845. doi: 10.1080/00222933.2015.1091101

[B12] Zheng ZM (1983) A new tetrigid species from China (Orthoptera: Tetrigidae). Acta Entomologica Sinica 26(1): 85–86. [In Chinese with English summary]

[B13] Zheng ZM (1996) Three new species of Tetrigidae from China. Acta Zootaxonomica Sinica 21(1): 83–88. [In Chinese with English summary]

[B14] Zheng ZM (2005) Fauna of Tetrigoidea from Western China. Science Press, Beijing, 501 pp. [In Chinese with English summary]

[B15] Zheng ZM Liang GQ (1991) On the genus *Thoradonta* Hancock from China (Orthoptera: Tetrigidae: Scelimeninae). Acta Entomologica Sinica 34(4): 453–457. [In Chinese with English summary]

